# Arbutin Inhibited Heat Stress-Induced Apoptosis and Promoted Proliferation and Migration of Heat-Injured Dermal Fibroblasts and Keratinocytes by Activating PI3K/AKT Signaling Pathway

**DOI:** 10.1155/2022/8798861

**Published:** 2022-09-15

**Authors:** Shugang Zhu, Zhen Yang, Lili Kong, Lijun Kong, Yuezhi Zhang

**Affiliations:** ^1^Department of Burn and Plastic Surgery, Yantai Affiliated Hospital of Binzhou Medical University, Shandong, China; ^2^Department of Biochemistry and Molecular Biology, Binzhou Medical University, Yantai, China; ^3^Department of Endocrinology and Metabolism, Yantai Affiliated Hospital of Binzhou Medical University, Shandong, China

## Abstract

**Objective:**

Studies have shown that arbutin has antioxidant and anti-inflammatory activities, which makes it suitable for treating skin wounds. We designed this study to investigate the effect of arbutin on heat-induced apoptosis, proliferation, and migration of dermal fibroblasts and keratinocytes and to explore the molecular mechanism.

**Methods:**

In vitro, HaCAT and dermal fibroblast (DFL) cells were cultured and used to establish a heat stress-injured skin cell model. We investigated the effects of arbutin on apoptosis, proliferation, and migration of HaCAT and DFL cells after heat stress injury. We then used immunoblotting to detect the expression of p-PI3K, PI3K, p-AKT, and AKT proteins for studying the underlying mechanisms and used a PI3K/AKT inhibitor (LY294002) to verify the efficacy of arbutin in HaCAT and DFL cells with heat stress injury.

**Results:**

Arbutin strongly inhibited heat stress-induced apoptosis, proliferation inhibition, and migration inhibition of HaCAT and DFL cells in vitro. Our results also showed that arbutin strongly decreased the ratio of Bax/Bcl2 protein expression and PCNA protein expression in HaCAT and DFL cells after treatment with heat stress. Furthermore, we also found that arbutin significantly increased the ratio of p-PI3K/PI3K and p-AKT/AKT protein expression, and LY294002 markedly reversed the effect of arbutin on heat stress-induced apoptosis, proliferation inhibition, and migration inhibition of HaCAT and DFL cells.

**Conclusion:**

Our finding indicated that arbutin inhibited heat stress-induced apoptosis and promoted proliferation and migration of heat-injured dermal fibroblasts and epidermal cells by activating the PI3K/AKT signaling pathway, suggesting that arbutin may provide an alternative therapeutic approach for the treatment of skin injury.

## 1. Introduction

The skin is the largest organ of the human body, accounting for about 14–17% of the human body weight, and has a series of physiological functions such as breathing, sensation, protection, absorption, secretion, excretion, temperature regulation, and metabolism, and it is also an important barrier to protect the homeostasis [1, 2]. In general, burns and scalds generally refer to skin and/or mucosal tissue damage caused by physical or chemical factors such as boiling water (oil), high-temperature gas, hot metal liquid or solid, electricity, flame, radiation, or chemical substances acting on the human body. In severe cases, deep tissues can also be injured [3, 4]. Burns and scalds are one of the most common accidental injuries in clinical practice, and about 20 million people suffer burns and scalds of varying degrees every year in China [5, 6].

Fibroblasts and keratinocytes are important components of dermal tissue, and they can promote skin wound healing through proliferation, migration, differentiation, secretion of multiple cytokines, and synthesis of multiple extracellular matrices, including collagen, when skin injury occurs [7, 8]. After skin injury, dermal fibroblasts and keratinocytes will first proliferate in mitosis. 4-5 days after a skin injury, they begin to synthesize and secrete extracellular matrix components (mainly collagen) and a variety of cytokines [7, 8]. This extracellular matrix and cytokines function with the new parenchyma capillaries to form granulation tissue, fill the wound, and provide a supporting structure for the proliferation, differentiation, growth, and adhesion of cells in the process of wound healing [9, 10]. Therefore, any drugs or alternative drugs that can change the proliferation, migration, biological state, and function of dermal fibroblasts and epidermal cells will affect the repair of skin damage.

Arbutin is a component extracted from bearberry leaves of the Rhododendron family and inhibits the activity of tyrosinase in the body and prevents the production of melanin, thereby reducing skin pigmentation and removing stains and freckles. It has antiseptic and anti-inflammatory effects and is mainly used in cosmetics [11, 12]. A recent study has found that arbutin can inhibit inflammation and inhibit cell apoptosis by activating the PI3K/AKT pathway [13], and an activated PI3K/AKT pathway has been confirmed to be related to cell survival, proliferation, and migration [14, 15]. However, the effect of arbutin on the healing of thermal burn skin wounds and their potential mechanisms are still not being studied. In this study, we established a heat stress-injured skin cell model in vitro using HaCAT and dermal fibroblast (DFL) cells to explore the effect of arbutin on heat stress-induced apoptosis, proliferation inhibition, and migration inhibition in HaCAT and DFL cells.

## 2. Materials and Methods

### 2.1. Cell Culture and Cell Treatment

HaCAT cells were purchased from the American type culture collection and dermal fibroblasts (DFL) cells were isolated from normal skin as previously described [16]. HaCAT and DFL cells were all cultured in DMEM medium (11965092, Gbico) supplemented with 10% foetal bovine serum (16140071, Gbico) at 37°C with 5% CO_2_. For heat stress, we seeded 1.5 × 10^6^ HaCAT or DFL cells/well in a 6-well cell culture plate; 12 hours later, cells were treated at 43°C for 50 min in a waterbath.

**Figure 1:**
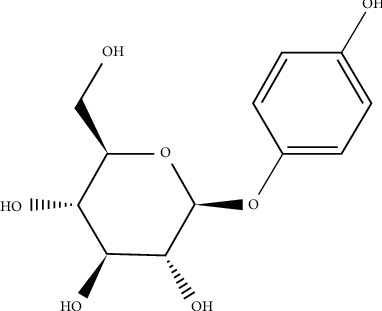
Molecular formula of arbutin.

After being treated with heat stress, we transferred cells to a normal environment (37°C and 5% CO_2_) for another 24 hours of cultivation. For the arbutin treatment group, 10, 50, and 100 *μ*mol/L arbutin (497-76-7, MERK) (Figure 1) was added into the cell culture medium. For the solvent group, the same volume of solvent as arbutin was added to the medium. For arbutin + LY294002 treatment, 100 *μ*mol/L arbutin (497-76-7, MERK) and 50 *μ*mol/L LY294002 (HY-10108, MedChemExpress) were added into the cell culture medium. HaCAT and DFL cells not treated at 43°C for 50 minutes were used as a control group.

### 2.2. Apoptosis Assay

We harvested cells to detect apoptosis using the Annexin V-FITC/PI apoptosis detection kit (40302ES20, YEASEN) as stated in the product manual. Briefly, we washed cells twice with precold PBS buffer, and then, 100 *μ*L binding buffer supplemented with 5 *μ*L Annexin V-FITC and 10 *μ*L PI staining solution was used to resuspend cells. After incubating at room temperature for 15 minutes in the dark, we used flow cytometry to detect cell apoptosis.

### 2.3. Western Blot

We lysed the cells to obtain the total protein using RIPA lysis buffer (R0013D, Beyotime), and then used a BCA kit to detect the total protein concentration. 50 *μ*g total protein was analyzed by 10% SDS-PAGE. After transferring to the PVDF membrane and sealing with 5% skimmed milk, the primary antibodies against Bax (ab32503, Abcam), Bcl2 (ab182858, Abcam), or PCNA (A12417, Abclone), or Ki-67 (ab1667, Abcam), or p-PI3K (ab278545, Abcam), or PI3K (ab140307, Abcam), or p-AKT (4060, Cell Signaling Technology), or AKT (4685, Cell Signaling Technology) were incubated overnight at 4°C. After being incubated with a secondary antibody at room temperature for 1 hour, the proteins were visualized with ECL solution (WBKLS0100, Beijing Xinjingke Biotechnologies Co., Ltd., China), followed by densitometry analysis using Image J 3.0 (IBM, USA). *β*-Actin was loaded as control.

### 2.4. Cell Proliferation Detection

We seeded 1 × 10^4^ cells into a 96-well cell culture plate. After being treated in different ways, we removed the cell medium and washed the cells 3 times with PBS. Then, 100 *μ*l cell culture medium supplemented with 10 ul CCK-8 solution (C0038, Beyotime) was added into cells to incubate for 1 hour at 37°C with 5% CO_2_. Finally, we detected OD450 to calculate the relative cell viability.

### 2.5. Immunofluorescence Staining of PCNA and Ki-67

1 × 10^4^ cells were seeded into the Lab-Tek cell culture well ((155411, Thermo Scientific). After being treated in different ways, we removed the cell medium and washed cells 3 times with PBS. After fixing cell with 4% paraformaldehyde for 10 minutes at room temperature, cells were blocked with 5% BSA for one hour at room temperature. Then, cells were incubated with PCNA-antibody (A12417, Abclone) or Ki-67-antibody (ab1667, Abcam) overnight at 4°C. The next day, we removed the primary antibody, and cells were incubated with Alexa Fluor488 goat-anti-rabbit IgG (H + L) (A11008, Invitrogen) after washing cells 3 times with PBS buffer. For cells, it should be counterstained, the nucleus with 5 *μ*g/mL DAPI for 5 minutes at room temperature. Finally, all samples were analyzed by confocal microscopy.

### 2.6. Transwell Assay for Determining Migration

After being treated in different ways, we harvested cells, and 0.5 × 10^5^ cells/100 *μ*l of medium were seeded into the upper chamber of transwell, and the lower chamber of the transwell is added with 600 *μ*L of McCoy's 5A medium containing 2.5% FBS. 48 hours later, we collected the lower chamber of the transwell and removed the cell medium. We washed cells 3 times with PBS. Finally, we stained cells with 0.1% crystal violet at room temperature for 20 minutes. After washing with PBS until there is no purple, we take pictures of the cells to count the number of cells under an optical microscope.

### 2.7. Statistical Analysis

GraphPad Prism 5 was used for statistical analysis in this study. Data were shown as (mean ± standard deviation), and the *P* value was calculated by one-way ANOVA. *P* < 0.05 indicated a significant difference.

## 3. Results

### 3.1. Arbutin Inhibited Heat Stress-Induced Apoptosis of Skin Cell

**Figure 2:**
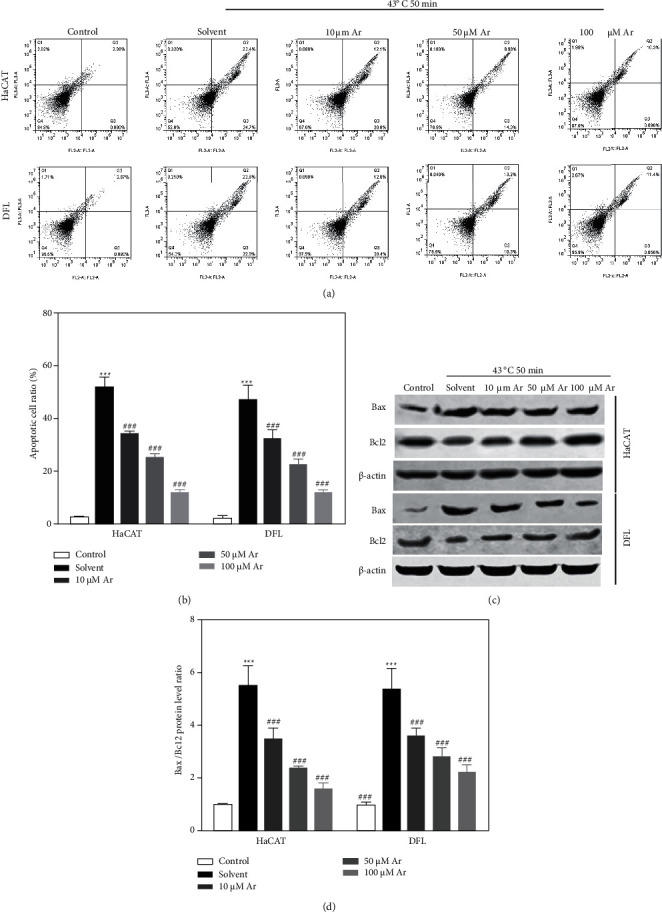
Effect of arbutin on heat stress-induced apoptosis of HaCAT and DFL cells. (a) Representative scatter plots of apoptosis in HaCAT and DFL cells detected by flow cytometry with solvent and different concentrations of arbutin (10, 50, and 100 *μ*mol/L) after 43°C-50 minutes heat stress. (b) Statistically compared proportion of apoptotic cells in (a) (*n* = 3). (c) Bax and Bcl2 protein expression in HaCAT and DFL cells of different groups detected by Western blotting. (d) Statistically compared expression of Bax and Bcl2 protein in (*n* = 3). *P* value was calculated by one-way ANOVA. ^*∗∗∗*^*P* < 0.001 vs. the control group; *P* < 0.001 vs. the solvent group.

To investigate the biological effect of arbutin on heat stress-induced apoptosis of skin cells, HaCAT and DFL cells were first treated at 43°C for 50 minutes and then transferred to a normal environment (37°C and 5% CO_2_) for cultivation with solvent or different concentrations of arbutin (10, 50, and 100 *μ*mol/L) for 24 hours. HaCAT and DFL cells not treated at 43°C for 50 minutes were used as control. As shown in Figures 2(a) and 2(b), the results of apoptosis detection by the flow cytometer suggested that compared with the control group, heat stress (43°C for 50 minutes) markedly increased the apoptosis of HaCAT and DFL cells without arbutin treatment (solvent group), while arbutin (Ar) treatment could significantly decrease the apoptosis of HaCAT and DFL cells induced by heat stress in a dose-dependent manner. In addition, we also detected the expression of apoptosis-related protein and found that heat stress significantly increased Bax protein expression and decreased Bcl2 protein expression, and arbutin (Ar) treatment could strongly decrease the evaluated Bax protein expression and increase the decreased Bcl2 protein expression induced by heat stress in HaCAT and DFL cells (Figure 2(c)). Compared with the control group, heat stress markedly increased the ratio of Bax/Bcl2 protein expression without arbutin treatment (solvent group), while Ar treatment could significantly decrease the ratio of Bax/Bcl2 protein expression in HaCAT and DFL cells induced by heat stress in a dose-dependent manner (Figure 2(d)).

### 3.2. Arbutin Inhibited Heat Stress-Induced Proliferation and Migration Inhibition in Skin Cell

**Figure 3:**
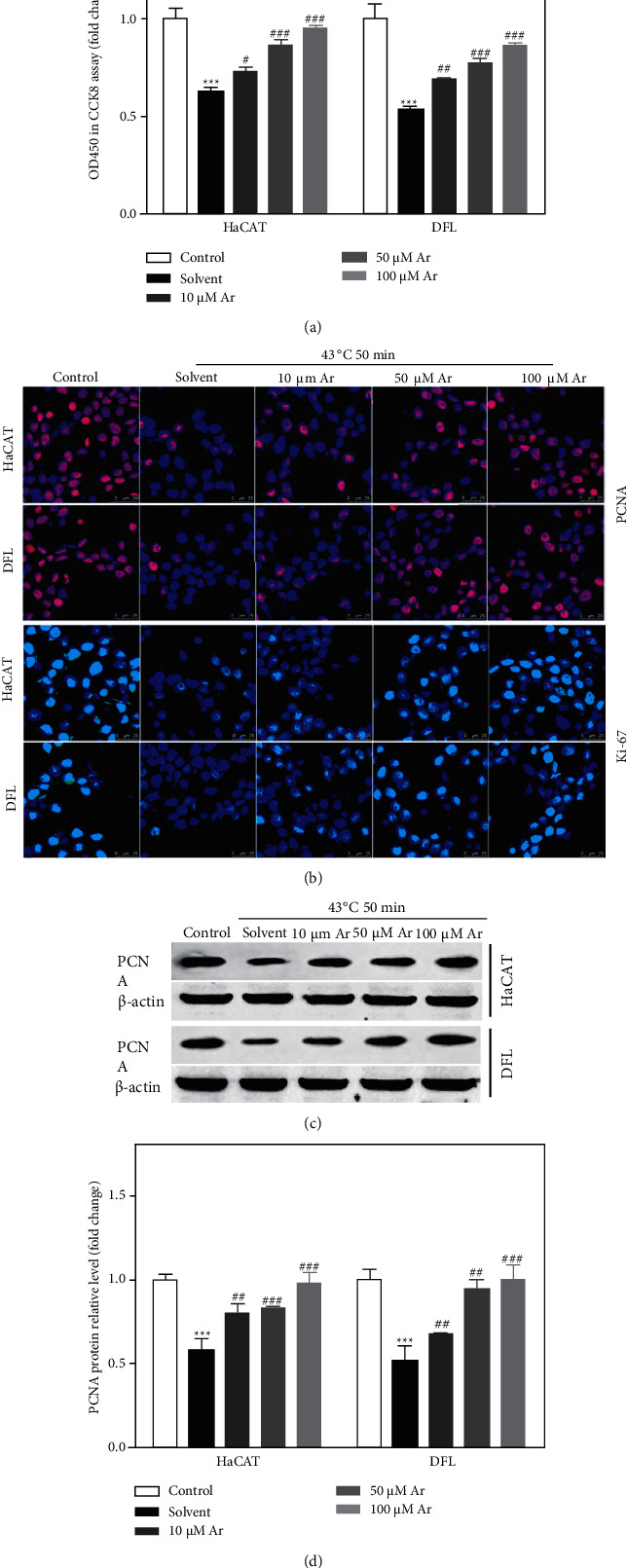
Effect of arbutin on the proliferation of HaCAT and DFL cells after induced by heat stress. (a) 48 hours after 4°C-50 minutes heat stress, the growth of HaCAT and DFL cells with solvent and different concentrations of arbutin (10, 50, and 100 *μ*mol/L) was observed; statistically compared the value of OD450 in the detection of CCK8 proliferation (*n* = 3). (b) The expression levels of PCNA and Ki-67 in HaCAT and DFL cells of different groups determined by immunofluorescence staining. (c) PCNA protein expression in HaCAT and DFL cells of different groups detected by Western blotting. (d) Statistically compared expression of PCNA protein (*n* = 3). *P* value was calculated by one-way ANOVA. ^*∗∗∗*^*P* < 0.001 vs. the control group; ^#^*P* < 0.05, ^##^*P* < 0.01, and ^###^*P* < 0.001 vs. the solvent group.

The proliferation and migration of skin cells are keys to skin wound healing. Herein, we studied the effect of arbutin on the proliferation of skin cell after treating with heat stress (43°C for 50 minutes) using CCK-8 assay. We added CCK-8 reagent to evaluate cell viability by detecting the absorbance at 450 nm (OD450) and found that the cell viability of HaCAT and DFL cells in the control group was significantly lower than that in the control group, and arbutin treatment could significantly decrease the viability of HaCAT and DFL cells induced by heat stress in a dose-dependent manner (Figure 3(a)). Moreover, we also detected the expression of proliferation-related proteins (such as PCNA and Ki-67) using cellular immunofluorescence. As shown in Figure 3(b), the expression of PCNA and Ki-67 protein in the solvent group was significantly lower than that in a control group, and arbutin treatment could significantly increase the expression of PCNA and Ki-67 protein in HaCAT and DFL cells induced by heat stress in a dose-dependent manner. Similarly, the results of immunoblotting showed that arbutin treatment could significantly increase the decreased expression of PCNA protein in HaCAT and DFL cells induced by heat stress in a dose-dependent manner (Figures 3(c) and 3(d)).

**Figure 4:**
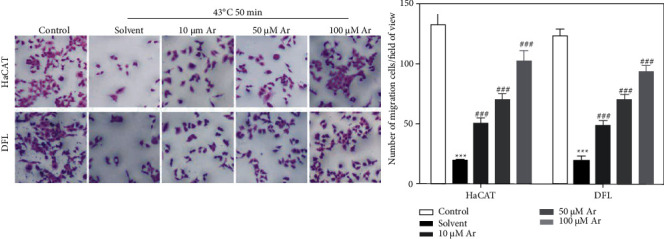
Effect of arbutin on the migration of HaCAT and DFL cells after induced by heat stress. Transwell chamber for determining the migration of HaCAT and DFL cells with solvent and different concentrations of arbutin (10, 50, and 100 *μ*mol/L) after 4°C-50 minutes heat stress (a). Statistically compared the number of cells that have migrated (*n* = 3) (b). *P* value was calculated by one-way ANOVA. ^*∗∗∗*^*P* < 0.001 vs. the control group; ^###^*P* < 0.001 vs. the solvent group.

A transwell chamber was used to assess the migration of HaCAT and DFL cells, and we found that the number of HaCAT and DFL cells migrated in the solvent group was significantly lower than that in the control group, and arbutin treatment could significantly increase the number of HaCAT and DFL cells migrated induced by heat stress in a dose-dependent manner (Figure 4).

### 3.3. Arbutin Activated PI3K/AKT Pathway in Heat-Injured Skin Cell

**Figure 5:**
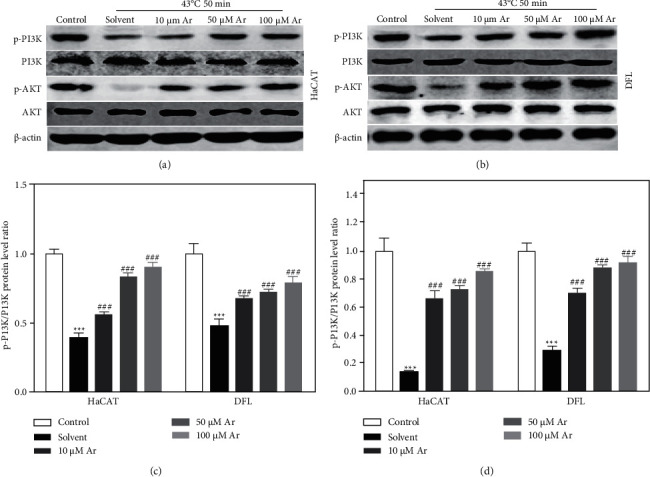
Effect of arbutin on the activation of the PI3K/AKT pathway in HaCAT and DFL cells after induced by heat stress. (a and b) Western blotting used to detect p-PI3K, PI3K, p-AKT, and AKT protein expressions in HaCAT (a) and DFL (b) cells with solvent and different concentrations of arbutin (10, 50, and 100 *μ*mol/L) after 4°C-50 minutes heat stress. (c) Statistically compared expression of p-PI3K/PI3K protein expression ratio in (a and b) (*n* = 3). (d) Statistically compared expression of p-AKT/AKT protein expression ratio in (a and b) (*n* = 3). *P* value was calculated by one-way ANOVA. ^*∗∗∗*^*P* < 0.001 vs. the control group; ^###^*P* < 0.001 vs. the solvent group.

Recently, a study found that arbutin could regulate inflammation and apoptosis by activating the PI3K/AKT pathway [13], and an activated PI3K/AKT pathway has been confirmed to be related to cell survival, proliferation, and migration [14, 15]. Therefore, we detected the expression of p-PI3K, PI3K, p-AKT, and AKT proteins in HaCAT and DFL cells. We found that there was no significant difference in the expression of PI3K and AKT protein in each group, while the expression of p-PI3K and p-AKT protein in HaCAT and DFL cells in the solvent group was significantly lower than that in the control group and arbutin treatment could significantly increase the expression of p-PI3K and p-AKT protein in HaCAT and DFL cells induced by heat stress in a dose-dependent manner (Figures 5(a) and 5(b)). Importantly, arbutin treatment significantly increases the decreased ratio of p-PI3K/PI3K and p-AKT/AKT protein expression in HaCAT and DFL cells induced by heat stress in a dose-dependent manner (Figures 5(c) and 5(d)).

### 3.4. Arbutin Inhibited Heat Stress-Induced Apoptosis of Skin Cell by Activating PI3K/AKT Pathway

**Figure 6:**
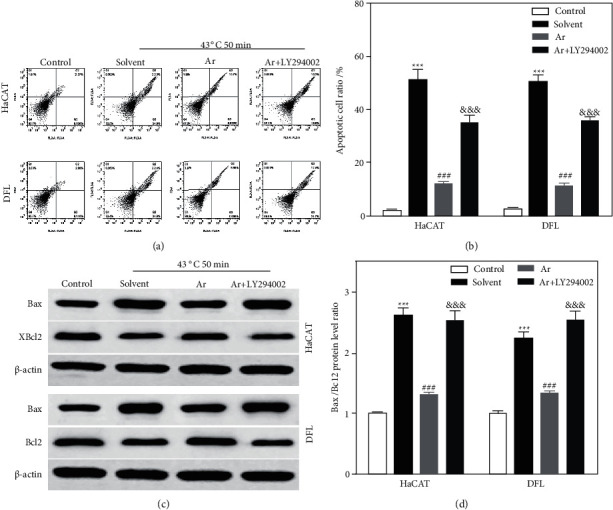
Arbutin inhibited heat stress-induced apoptosis of HaCAT and DFL cells by activating the PI3K/AKT pathway. (a) Representative scatter plots of apoptosis in HaCAT and DFL cells detected by flow cytometry with solvent, 100 *μ*mol/L arbutin, and arbutin (100 *μ*mol/L) + LY294002 (50 *μ*mol/L). (b) Statistically compared proportion of apoptotic cells in (a) (*n* = 3). (c) Bax and Bcl2 protein expressions in HaCAT and DFL cells of different groups detected using Western blotting. (d) Statistically compared the expression of Bax and Bcl2 protein in (c) (*n* = 3). *P* value was calculated by one-way ANOVA. ^*∗∗∗*^*P* < 0.001 vs. the control group; ^###^*P* < 0.001 vs. the solvent group; ^&&&^*P* < 0.001 vs. the Ar group.

To investigate whether arbutin inhibited heat stress-induced apoptosis of skin cells by activating the PI3K/AKT signaling pathway, we used LY294002, an inhibitor of PI3K/AKT, to inhibit the activation of the PI3K/AKT signaling pathway. As shown in Figures 6(a) and 6(b), 100 *μ*mol/L Ar could significantly decrease heat stress-induced apoptosis of HaCAT and DFL cells, while the simultaneous treatment with LY294002 reversed this effect. At the same time, the results of immunoblotting showed that 100 *μ*mol/L Ar significantly decreased the ratio of Bax/Bcl2 protein expression in HaCAT and DFL cells being treated with heat stress, while the simultaneous treatment with LY294002 reversed this effect (Figures 6(a) and 6(d)).

### 3.5. Arbutin Relieved Proliferation and Migration Inhibition in Heat-Injured Skin Cell by Activating PI3K/AKT Pathway

**Figure 7:**
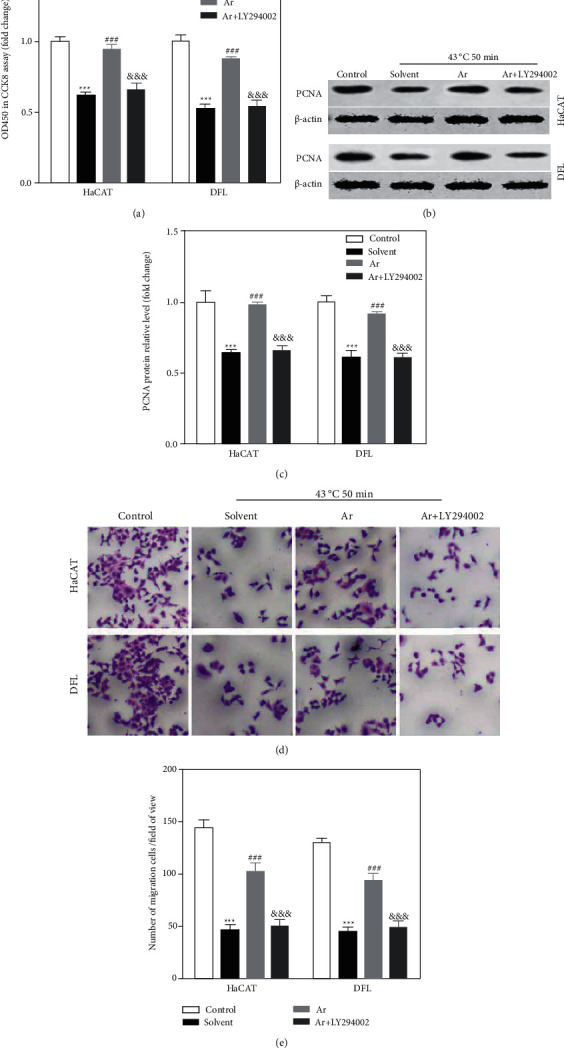
Arbutin promoted the proliferation and migration of HaCAT and DFL cells after induced by heat stress by activating the PI3K/AKT pathway. (a) HaCAT and DFL cells treated with solvent, 100 *μ*mol/L arbutin (Ar), and arbutin (100 *μ*mol/L) + LY294002 (50 *μ*mol/L) for 48 hours after 43°C-50 min heat stress; statistically compared the value of OD450 in the detection of CCK8 (*n* = 3). (b) PCNA protein expression in HaCAT and DFL cells of different groups detected by Western blotting. (c) Statistically compared expression of PCNA protein in (b) (*n* = 3). (d) Transwell chamber for determining the migration of HaCAT and DFL cells in different groups. (e) Statistically compared the number of cells that have migrated in (d) (*n* = 3). *P* value was calculated by one-way ANOVA. ^*∗∗∗*^*P* < 0.001 vs. the control group; ^###^*P* < 0.001 vs. the solvent group; ^&&&^*P* < 0.001 vs. the Ar group.

After being heat stressed, we transferred to a normal environment (37°C and 5% CO_2_) for cultivation with solvent or 100 *μ*mol/L arbutin (Ar) or arbutin (100 *μ*mol/L) + LY294002 (50 *μ*mol/L) for 48 hours and then assessed the proliferation of HaCAT and DFL cells using CCK-8 assay. The results of CCK-8 assay also showed that 100 *μ*mol/L arbutin significantly increased the viability of HaCAT and DFL cells, which was compared with solvent group, while the simultaneous treatment with LY294002 reversed this effect (Figure 7(a)). In addition, we also found that arbutin significantly increased the expression of PCNA protein in HaCAT and DFL cells after being treated with heat stress, while LY294002 significantly decreased the evaluated PCNA protein expression induced by arbutin in HaCAT and DFL cells after being treated with heat stress (Figures 7(b) and 7(c)). At the same time, we also investigated whether arbutin relieved migration inhibition in heat-injured skin cell by activating the PI3K/AKT pathway using a transwell chamber and found arbutin treatment significantly promoted the migration of HaCAT and DFL cells, while the simultaneous treatment with LY294002 reversed this effect (Figures 7(d) and 7(e)).

## 4. Discussion

With the development of social industrialization and the improvement of people's living standards, the incidence of burns and scalds is also increasing [17, 18]. At present, there are still four difficulties in the clinical treatment of burns and scalds: “wound pain, progressive necrosis, susceptibility to infection, and scar healing” [19, 20]. Therefore, how to improve the therapeutic effect of burns and scalds, accelerate the healing process of scalds, and inhibit the formation of scars is one of the keys and most difficult points of clinical research today. In this study, we first found that arbutin inhibited heat stress-induced apoptosis of HaCAT and DFL cells in vitro. Apoptosis plays an important role in the early progressive deepening of scald wounds, and the increase in apoptosis rate aggravates the oxidative damage of the wound and deepens the wound [21, 22]. Therefore, arbutin may provide an alternative therapeutic approach for the treatment of skin injury by inhibiting heat stress-induced apoptosis.

Skin burn repair is a very complex physiological and pathological process, which mainly includes several stages of inflammation, cell proliferation, connective tissue formation, wound contraction, and wound remodeling [23, 24]. While, it is clear that a series of activities of repairing cells are the basis of wound repair, and fibroblasts and keratinocytes are the most important repair cells in wound repair [25, 26]. Keratinocytes are the main constituent cells of the epidermis, accounting for more than 80% of epidermal cells, and the migration, proliferation, and differentiation of keratinocytes to form a new epidermal layer is one of the necessary processes to cover the wound, and it is also an important sign of wound healing [27, 28]. Fibroblasts are resident cells in the dermis and play a key role in wound repair through migration, proliferation, differentiation, and the secretion of collagen and cytokines during wound healing and the development of fibrosis [29, 30]. At the same time, fibroblasts are also closely related to the formation of extracellular matrix, granulation tissue, and scar tissue [29, 30].

In this study, the results showed that arbutin strongly relieved heat stress-induced proliferation inhibition and migration inhibition of HaCAT and DFL cells in vitro. Nasiri et al. [31] used silver sulfadiazine as a positive control drug to study the effect of mallow extract on deep second-degree scalds in rats, and the results showed that mallow extract can significantly promote the proliferation of fibroblasts and promote wound vascular regeneration. In addition, Mehrabani et al. [32] found that external application of curcumin could significantly promote the proliferation of epidermal cells and re-epithelialization of the wound surface, and the structure and function of the epidermis would be complete after healing. Combined with our research, it suggests that arbutin may provide an alternative therapeutic approach for the treatment of skin injury by relieving heat stress-induced proliferation inhibition and migration inhibition of fibroblasts and keratinocytes.

Phosphatidylinositol 3-kinase (PI3K) is a kinase that specifically catalyzes the phosphorylation of the 3-position hydroxyl group of phosphatidylinositol (PI) to produce a second messenger function, and serine/threonine protein kinase (Akt) is at the central link of the PI3K/Akt pathway and plays an important biological role in cell apoptosis, survival, proliferation, and other activities [33, 34]. In the study of wound healing, previous studies have found that wound healing is the result of the combined effects of many growth factors, and the effects of various growth factors are mostly achieved through the PI3K/Akt signaling pathway [35, 36]. The activation of the PI3K/Akt pathway by different growth factors can cause different downstream. The participation of signal molecules can play a different role in promoting healing [35, 36]. Importantly, a previous study has found that the PI3K/Akt signaling pathway is the target of arbutin, and arbutin attenuates LPS-induced acute kidney injury by inhibiting inflammation and apoptosis via the PI3K/Akt/Nrf2 pathway [13].

In the present study, we first investigated the effect of arbutin on the activation of PI3K/Akt in HaCAT and DFL cells after being treated with heat stress and found that arbutin activated the PI3K/AKT pathway in heat-injured skin cells. Previous studies found that activating the PI3K/AKT signaling pathway will help fibroblasts and keratinocytes to survive from heat stress injury and can promote their proliferation and migration [7,8], and the PI3K/AKT pathway is a key target to promote wound healing [35, 36]. Hosotani et al. found that IL-18 inhibited the apoptosis of normal human neonatal foreskin epidermal keratinocytes (NHEK-F) by activating the PI3K/AKT signaling pathway [37]. Moreover, the activation of the PI3K/AKT pathway has been found to promote the proliferation and migration of keratinocytes, such as, Chen et al. found that miRNA6 enhanced viability, colony formation, and migration of keratinocyte HaCaT cells by activating the PI3K/AKT signaling pathway [38]. The PI3K/AKT signaling pathway could also be used as a drug target to promote keratinocyte proliferation [39]. Similarly, the activation of the PI3K/AKT signaling pathway has not only been found to enhance the antiapoptotic ability of fibroblasts [40] but also promote the proliferation and migration of fibroblasts [41, 42]. Herein, we used LY294002 to inhibit the activation of the PI3K/AKT signaling pathway and found that LY294002 reversed these effects of arbutin on HaCAT and DFL cells after being treated with heat stress, suggesting arbutin inhibited heat stress-induced apoptosis, proliferation inhibition, and migration of skin cells by activating the PI3K/AKT signaling pathway.

All in all, our finding suggests that arbutin is a potential drug for treating burns, and its mechanism is that arbutin inhibited heat stress-induced apoptosis and promoted proliferation and migration of heat-injured dermal fibroblasts and epidermal cells by activating the PI3K/AKT signaling pathway. However, deletion of PI3K/AKT pathway activators is insufficient for understanding the mechanism of arbutin in the treatment of scalds.

## Data Availability

The data used to support the findings of this study are available from the corresponding author upon request.
